# Renal Artery Stenosis and Ipsilateral Renal Cell Carcinoma: Description of an In Situ Partial Nephrectomy and Splenorenal Arterial Bypass

**DOI:** 10.1100/tsw.2004.83

**Published:** 2004-06-07

**Authors:** Scott A. Slavis, George S. Ganesan, Claudia Swift, Ronald C. Ruppert

**Affiliations:** Department of Urology and Renal Transplantation, Sunrise Hospital, Las Vegas, Nevada, USA

## Abstract

A case of a renal artery stenosis and ipsilateral renal cell carcinoma with long term results is reported. A 65-year-old man with renovascular hypertension, renal insufficiency, and nephrotic range proteinuria presented with an incidental renal cell carcinoma. Concomitant in situ left partial nephrectomy and splenorenal arterial bypass was achieved. The patient is doing well without evidence of malignancy, stable renal function, markedly improved proteinuria and stable blood pressure more than three years later. The techniques of this procedure are detailed and underscore the possibility of successful removal of a renal cell carcinoma with preservation of renal function despite renal artery stenosis.

## INTRODUCTION

Renal parenchymal sparing is widely accepted for the treatment of renal cell cancer particularly in patients with renal insufficiency.[1]Likewise, the benefits of renal revascularization procedures are well documented.[2,3] These techniques individually have been well elucidated.[4] However, we believe this is the first detailed report of a simultaneous ipsilateral partial nephrectomy and renal artery bypass procedure.

## CASE REPORT

A 65-year-old man presented with long-standing hypertension, renal insufficiency, nephrotic range proteinuria and transient ischemic attacks. In the course of evaluation for a possible abdominal aortic aneurysm and hypertension, screening abdominal ultrasound revealed an incidental two centimeter solid left renal mass. His antihypertensive medications were nifedipine (Procardia XLTM)* 90 mg. per day and clonidine patch 0.1 mg./day/week. Blood pressure was 150/90 mm. Hg. and serum creatinine was 2.3 mg.%. Creatinine clearance was 49 ml. per minute and 24 hour urinary protein was 2.8 grams. CT scan confirmed a 2 cm. solid left renal mass (fig.1a). The CT also showed well perfused kidneys with equal

*Pfizer Inc., New York.

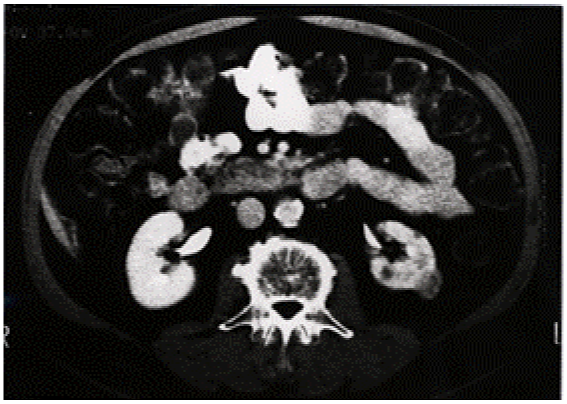

**FIGURE 1a.** Preoperative CT revealing 2 cm. solid lower pole left renal mass.

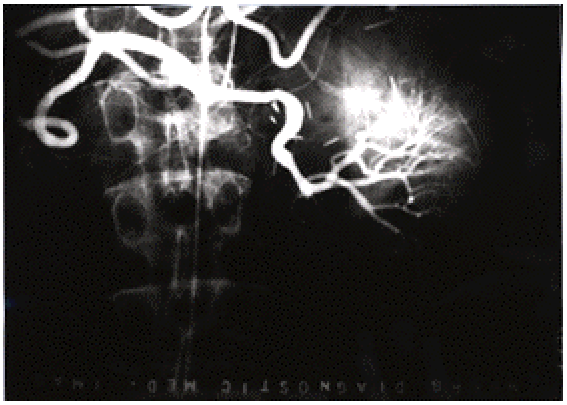

**FIGURE 1b**. Preoperative angiogram showing high grade left renal artery ostial stenosis (arrow).

dimensions. A differential nuclear renal function study was not considered necessary. Metastatic workup including CT of the chest, isotopic bone scan, and MRI of the abdomen were unremarkable. Renal angiography showed a hypervascular left lower pole renal mass, high grade ostial stenosis of a single left renal artery, and a normal right renal artery (fig.1b). The abdominal aorta and iliac arteries demonstrated moderate calcificatherosclerosis, minimal ostial disease of the celiac takeoff and a patent splenic artery. Further testing demonstrated no evidence of significant carotid or coronary artery disease.

Because of the patients renal insufficiency and a well perfused, viable left kidney a partial nephrectomy and splenorenal arterial bypass was selected as the procedure of choice. On July 9, 1994, exploration was performed through a left subcostal intraperitoneal approach. After ensuring there were no metastatic deposits, the left retroperitoneum was exposed by reflecting the descending and transverse colon medially. With the exception of the kidney’s upper pole where the adrenal was dissected free, Gerota’s fascia was retained on the kidney and its mass. Next, the main renal artery and vein were completely mobilized. Generally, when performing partial nephrectomy the renal artery is occluded with a vascular clamp and the kidney is cooled with sterile ice slush to limit blood loss and acute tubular necrosis. However, owing to the high grade stenosis of the renal artery with its consequent increased risk of thrombosis, we elected not to clamp the artery and perform partial nephrectomy without cooling. Systemic heparinization was not employed. The tumor and overlying perinephric fat were excised with sharp dissection. Intra operative pathology consultation demonstrated 0.5 cm. tumor-free parenchymal margins. Bleeding points and open collecting system was controlled with absorbable sutures. The renal capsule and a portion of adjacent remaining vascularized perinephric fat were re-approximated over the defect created by removal of the tumor. After ensuring good hemostasis and doppler pulsations to the remaining renal tissue, attention was focused on the revascularization procedure.

The splenic artery was mobilized from near the celiac axis to the tail of the pancreas. Small branches were divided between fine silk ties. Avascular bulldog clamp was placed proximally and the splenic artery was transected and secured distally with a heavy silk tie. Good blood flow was demonstrated by temporarily releasing the clamp. At this juncture the left renal artery was secured proximally with a heavy silk tie and divided just distal to the area of ostial stenosis. It was flushed with heparinized saline and a small vascular clamp was placed on the distal renal artery. Both the cut ends of the renal and splenic arteries were trimmed to length and spatulated. The vessels were then anastomosed end to end using fine interrupted cardiovascular sutures. The bulldogs were released, first from the renal artery and then from the splenic artery. There was an excellent pulse in the renal artery and no anastomotic leakage. The vessels had a smooth course without kinking, tension, or coiling. Prior to closing, a closed suction drain was employed in the perinephric space to identify the development of any possible urinoma.

Postoperative course was marred by gangrenous cholecystitis and recurrent aspiration from a poor gag reflex. However, complete recovery ensued. Final pathologic staging confirmed clear parenchymal margins but there were microscopic foci of tumor in the perirenal fat. The tumor had clear cell pattern and was moderately differentiated with a nuclear grade of II of IV. Also, the surrounding noncancerous renal parenchyma showed 30% glomerulosclerosis and cholesterol emboli. The patient is now doing well more than three years later. Angiography shows excellent restoration of blood flow to the kidney (fig. 2). There is no evidence of any recurrent renal cell carcinoma on serial CT scans and chest X-rays. Blood pressure is better controlled (135/82 mm./Hg.) on nifedipine (Procardia XL) 30 mg. per day, clonidine 0.1 mg. per day, and atenolol25 mg. bid. Serum creatinine is stable and measures 1.9 mg.%, creatinine clearance is 34.1 ml. per minute, and 24 hour urinary protein is 0.48 grams.

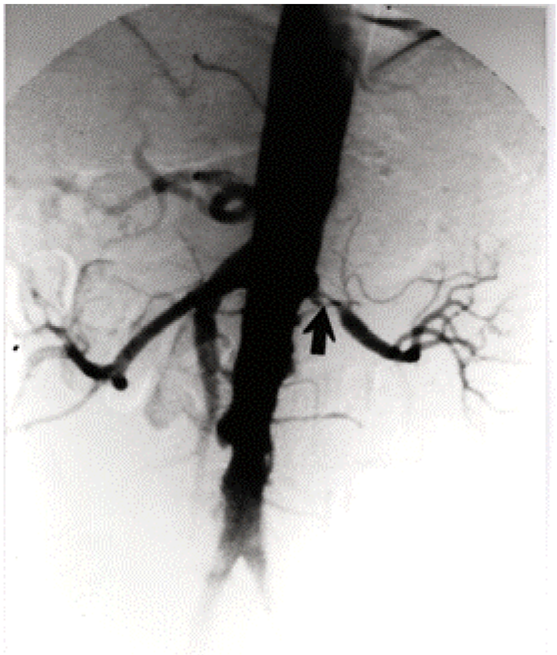

**FIGURE 2**. Postoperative angiogram showing widely patent end to end splenic artery to left renal artery bypass.

## DISCUSSION

Renal cell carcinoma and coexistent ipsilateral renal artery stenosis appear to be an uncommon combination. Kaufman et. al.[5] described two patients with partial nephrectomy and vascular repair. However, details are not complete. Dean et. al.[6] presented two cases of partial nephrectomy and autotransplantation for renal cell carcinoma and renal artery stenosis. Campbell et. al.[7] presented 4 patients with partial nephrectomy and ipsilateral renal artery bypass; 3 with aortorenal bypass and one with hepatorenal bypass.

One goal of therapy should include preservation of renal parenchyma when there is potential for risk for ensuing renal failure and need for dialysis. Our patient had a creatinine clearance of 49 ml./min. suggesting the need for nephron sparing surgery. Another goal of therapy is the correction of significant renal artery stenosis to preserve functioning renal tissue, improve or stabilize blood pressure control and correct ischemic related proteinuria. The association of ischemic nephropathy and proteinuria is well documented and may be corrected by reversing the problem.[8] Our case further documents this connection.

One consideration when performing nephron sparing surgery includes the possibility of local recurrence either from multifocality, tumor spillage or inadequate margins. Fortunately, the incidence is generally only 4%.[9] Certainly in our case the expected recurrence would be higher (8.2%)[9] as there was perinephric fat involvement with tumor. Close follow up has not shown any recurrence but periodic, lifelong imaging is mandatory.

Careful preoperative preparation is mandatory to avoid significant cardiovascular or cerebrovascular accidents. Furthermore, precise surgical planning is needed to select the appropriate revascularization procedure most feasible. Our patient had an abdominal aortogram, in the anterior-posterior view as well as the lateral view. The lateral view enables good visualization of the celiac axis origin. The primary open surgical treatment option for left sided renovascular disease would be aortorenal bypass with an autologous graft with saphenous vein or internal iliac artery. If the aorta is significantly diseased then the next option would be a left iliac artery to left renal artery bypass with saphenous vein. Unfortunately, our patient had both aortic and iliac artery atherosclerosis. The celiac axis was normal indicating a normal splenic artery without significant obstruction. Therefore, splenorenal arterial bypass was chosen.

In conclusion, we have shown excellent results in a patient requiring ipsilateral simultaneous partial nephrectomy and splenorenal arterial bypass. There is no evidence of recurrent malignancy as well as stable blood pressure control and renal function for more than three years.
